# Integration of Abscisic Acid Signaling with Other Signaling Pathways in Plant Stress Responses and Development

**DOI:** 10.3390/plants8120592

**Published:** 2019-12-11

**Authors:** Manu Kumar, Mahipal Singh Kesawat, Asjad Ali, Sang-Choon Lee, Sarvajeet Singh Gill, Hyun Uk Kim

**Affiliations:** 1 Department of Bioindustry and Bioresource Engineering, Plant Engineering Research Institute, Sejong University, Seoul 05006, Korea; 2 Department of Agriculture, Sri Sri University, Cuttack 754-006, India; kesawatbsmahi@gmail.com; 3 Southern Cross Plant Science, Southern Cross University, East Lismore NSW 2480, Australia; asjaddu@gmail.com; 4 Phyzen Co, Seongnam 13558, Korea; sclee0923@phyzen.com; 5 Stress Physiology and Molecular Biology Lab, Centre for Biotechnology, MD University, Rohtak 124001, India; ssgill14@mdurohtak.ac.in

**Keywords:** abscisic acid, abiotic stress signaling, ubiquitination, seed germination, E3 ubiquitin ligase, stomatal regulation

## Abstract

Plants are immobile and, to overcome harsh environmental conditions such as drought, salt, and cold, they have evolved complex signaling pathways. Abscisic acid (ABA), an isoprenoid phytohormone, is a critical signaling mediator that regulates diverse biological processes in various organisms. Significant progress has been made in the determination and characterization of key ABA-mediated molecular factors involved in different stress responses, including stomatal closure and developmental processes, such as seed germination and bud dormancy. Since ABA signaling is a complex signaling network that integrates with other signaling pathways, the dissection of its intricate regulatory network is necessary to understand the function of essential regulatory genes involved in ABA signaling. In the present review, we focus on two aspects of ABA signaling. First, we examine the perception of the stress signal (abiotic and biotic) and the response network of ABA signaling components that transduce the signal to the downstream pathway to respond to stress tolerance, regulation of stomata, and ABA signaling component ubiquitination. Second, ABA signaling in plant development processes, such as lateral root growth regulation, seed germination, and flowering time regulation is investigated. Examining such diverse signal integration dynamics could enhance our understanding of the underlying genetic, biochemical, and molecular mechanisms of ABA signaling networks in plants.

## 1. Introduction

Abscisic acid (ABA) signaling (perception, signaling, and tolerance) in plants is a complex response for which there are considerable knowledge gaps at the molecular level. ABA is a plant phytohormone with a small lipophilic sesquiterpenoid (C15) structure [1]. It has a key role in stress adaptation in addition to being critical in numerous biological processes, such as bud dormancy and seed germination [2,3,4,5,6]. In the 1960s, pioneering studies on ABA (initially termed “abscisin” and “dormin”) reported that it was accumulated in immature cotton balls that succumbed to ethylene-triggered abscission and over-wintering buds [4,7,8]. Later, it was demonstrated that under such conditions and developmental stages, plants were experiencing drought stress [5,9,10,11,12,13,14,15,16,17]. Therefore, ABA is a misnomer [18], even though it plays a role in leaf senescence and seed dormancy, potentially via osmotic effects [19,20,21]. It has been observed that drought-stressed vegetative tissues of numerous plants accumulate ABA (40-fold induction) within hours of osmotic stress and then it decreases after rehydration. In addition, ABA has been considered a long-distance stress signal between shoots and roots [22]. Therefore, the study of spatiotemporal expression of genes that control ABA metabolism’s rate-limiting steps is essential for understanding how plants adapt to stress. Other than its role in adaptation to abiotic stress, ABA has been shown to be a key regulator of pathogen virulence [23,24,25,26,27], which could offer insights into the basis of the ABA-synthesizing ability of numerous bio- and necrotrophic microbes [24,28,29,30].

Gene products acting in the vicinity of the cell wall or at the interface of the plasma membrane/cytoskeleton/cell wall are considered the most likely elements to participate in initial stress perception. For instance, gated aquaporins (plasma membrane intrinsic proteins (PIPs)) and osmo-/ion channels at the cell wall–plasma membrane interface may be implicated in the upstream perception [31,32,33]. The receptor of ABA remained unknown until 2009. Before then, several ABA receptors had been reported [34,35,36,37,38,39,40]; however, further investigations did not substantiate any of them. In 2009, two independent studies reported the steroidogenic acute regulatory protein (StAR)-related lipid-transfer (START) domain of the significant Bet v1 (birch pollen allergen) superfamily of proteins as candidate ABA receptors [41,42,43,44]. All 14 members of the protein family are called regulatory components of ABA receptor, RCAR1–RCAR14, [41], or pyrabactin resistance 1 (PYR1) and PYR1-like 1–13 [42]. The discovery of PYR1-like components (PYLs) laid the foundation for the unraveling of the ABA signaling mechanism in detail. Such findings opened the door for advancements in the ABA signaling field and were appropriately recognized as scientific breakthroughs of the year [45,46]. Multiple structure studies have clarified the interactions at the molecular level comprising a signaling cascade consisting of the PYL ABA receptors, the core ABA signaling pathway, Snf1-related protein kinases 2 (SnRK2s), and type 2C protein phosphatases (PP2Cs). ABA binding induces PYL protein interaction with the active site of PP2C and inhibits phosphatase activity by blocking the PP2C catalytic site (SnRK2 substrate) [47,48,49,50,51]. Such findings shed light on the ABA signaling transduction pathway, which could facilitate the unraveling of abiotic stress tolerance as well as various developmental processes in plants.

Numerous reviews have explored the specific aspects of ABA responses in detail [42,52,53,54,55,56,57,58,59,60], including the relationship between ABA signaling and abiotic stress responses, calcium signaling, MAPK signaling, and ubiquitination. In addition, abiotic stress tolerance has been reviewed extensively, although with less emphasis on seed development and lateral root formation, and no reviews have focused on the overall ABA signaling network [42,52,53,54,55,56,57,58,59,60]. ABA signaling is a complex network that works in tandem with other signaling pathways. Therefore, it is important to present an overall network taking into account recent advancements to fill the gaps that have not been addressed to date. In this review, we integrated signaling pathways that align with the ABA signaling pathway, which displays a complex network, being active during both plant abiotic stress tolerance and plant development. We also added recent findings to the existing ABA signaling network in plants.

## 2. Ubiquitination in ABA Signaling

**Figure 1:**
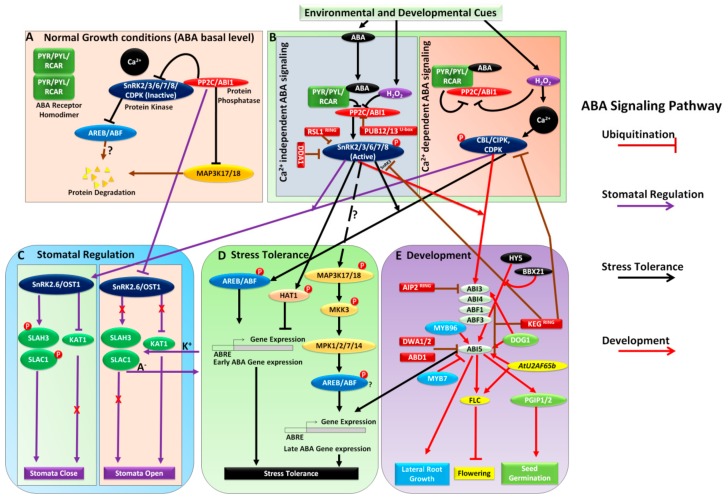
Overview of the abscisic acid (ABA) signaling pathway. (**A**) Inactivation of SnRKs, CIPKs, and CDPKs under normal growth conditions (light orange box). PP2C (red oval box) plays an important role in the inactivation of SnRKs, CIPKs, and CDPKs. Inactive MAP3K17/18 (orange oval box) and AREB/ABF (Abscisic Acid Response Element/Abre-Binding Factor) (yellow oval box) undergo protein degradation. (**B**) Initial perception of environmental and developmental cues. ABA signaling is transduced in Ca^2+^-independent (light blue box) as well as Ca^2+^-dependent (light orange box) manners. Active SnRKs, CIPKs, and CDPKs (dark blue oval box) play important roles in downstream signal transduction. (**C**) Stomatal regulation via ABA signaling in response to stress and healthy conditions. Under stress conditions, stomatal regulation (purple arrow →) is carried out by active SnRK2.6/OST1 (blue oval box) through the regulation of downstream ion channel genes (green oval boxes), such as *SLAH3*, *SLAC1*, and *KAT1*. This regulation helps stomata remain closed to avoid loss of excessive water under adverse conditions. Under normal conditions, SnRK2.6/OST1 inactivated by PP2C cannot regulate the downstream genes; thus, stomata remain open. (**D**) Response to stress tolerance via the ABA signaling pathway. The stress tolerance mechanism (black arrow →) is regulated in Ca^2+^-independent as well as Ca^2+^-dependent manners. The MAP kinase cascade (orange oval box) pathway carries the signal for the response to abiotic stress tolerance. It delays ABA gene expression. Contrarily, signal transduction via only AREB/ABF (yellow oval box) shows early expression of ABA related genes, resulting in an early response to stress tolerance. (**E**) Involvement of ABA signaling in the plant developmental process. Downstream ABA signaling involved in different developmental processes (red arrow →) such as seed germination (light green oval and square boxes), lateral root growth (light blue oval and square boxes), and regulation of flowering time (yellow oval and square boxes). ABI5 emerges as a critical ABA signaling component in the regulation of the plant developmental process. ABA signaling integrates with light signaling (black dark oval box) to regulate plant development. The brown tack facing up (⊥) indicates the role of ubiquitination in ABA signaling. These E3 ubiquitin ligase elements in ABA signaling guide the inactive protein to undergo degradation. The question mark (?) indicates the unknown pathway.

**Figure 2:**
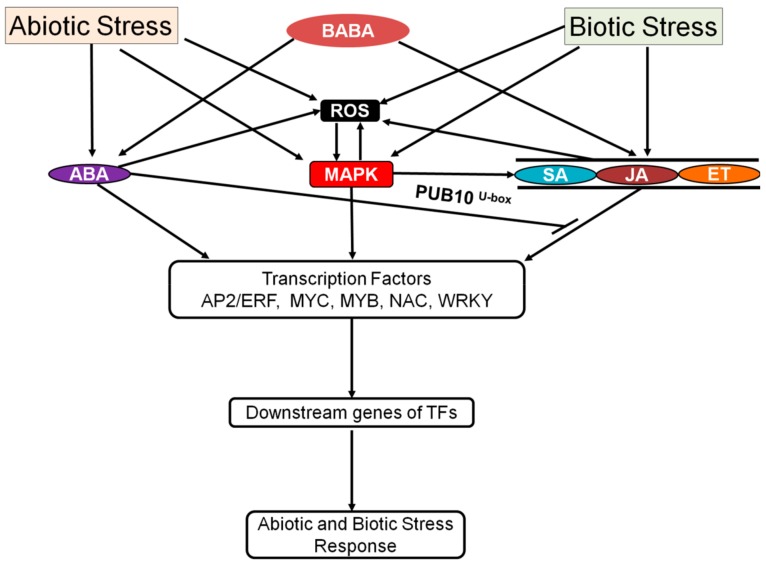
A simplified schematic diagram showing synergistic and antagonistic interactions between the ABA signaling pathway and other hormonal signaling pathways during abiotic and biotic stress.

Protein post-translational modification by ubiquitination has been studied during various aspects of stress responses, plant development, and growth [61,62]. Since ABA is a major phytohormone and plays a vital role in plant growth and stress responses, the regulation of its signaling components must be subjected to ubiquitination. Reports have emerged regarding E3 ligase-mediated ubiquitination of ABA signaling components [60]. The E3 ubiquitin ligases discussed in this review are indicated using dark red square boxes in Figure 1B,E. ABA receptors (PYR/PYL/RCAR) in plants are regulated by degradation via the ubiquitin-26S proteasome system. Damaged DNA binding protein 1 associated 1 (DDA1) from Cul4-based E3 ligase complexes and a single subunit, the RING-type E3 ligase RING FINGER OF SEED LONGEVITY1 (RSL1), are involved in the process [63,64], which suggests that, in the ABA signaling pathway, RSL1 acts as the negative regulator by regulating the ABA receptor through ubiquitination. Two plant U-box protein family members, PUB12 and PUB13, interact with ABI1. *ABI1* is induced in *pub12*/*pub13* mutants compared within wild types irrespective of ABA presence; however, it can ubiquitinate ABI1 only in the presence of both PYR1 and ABA, which indicates that the interaction between PYR1 and ABI promotes ABI1 degradation by PUB12/13 [65]. An E3 ubiquitin ligase, PLANT U-BOX PROTEIN10 (PUB10), modulates ABA signaling in Arabidopsis. *PUB10-OX* plants phenocopied *myc2*, whereas the *pub10* plants phenocopied *MYC2-OX* plants in response to ABA, indicating the regulation of MYC2 (a jasmonic acid (JA) signaling component) by PUB10 (Figure 2) [66]. A KEEP ON GOING (KEG) E3 ligase with a truncated RING domain also acts as a bait for the CIPK26 interaction because it acts as the negative regulator in ABA signaling [67,68]. ABA also induces the degradation of KEG by self-ubiquitination, resulting in the accumulation of ABI5 [69]. KEG also ubiquitinates and degrades ABF1 and ABF3 by interacting directly with them [70]. The results of the studies above suggested that ABF1, ABF3, and ABI5 were the substrates for E3 ligase KEG.

ABD1, DWA1, and DWA2, which are associated with Cul4-based E3 ubiquitin ligases, were reported to be responsible for ABA signaling through the degradation of ABI5 by regulated ubiquitination in the nucleus via the ubiquitin-26S proteasome system [71,72,73]. Single mutants, *abd1*, *dwa1*, and *dwa2*, and a double mutant, *dwa1/dwa2*, display ABA-hypersensitive phenotypes during seed germination and seedling growth [72,73], which indicates ABI5 acts as a target for ABD1, DWA1, and DWA2, Cul4-based E3 ubiquitin ligases, which leads to the negative regulation of ABA signaling in the nucleus. ABI3 INTERACTING PROTEIN2 is a functional RING-type E3 ligase that interacts with an unstable protein, ABI3, and is degraded via the ubiquitin-26S proteasome system [74]. Different types of E3 ligases with dual roles have been reported participating in the regulation of ABA signaling; however, the knowledge about their substrates and studies related to their association with ABA signaling is an ongoing process.

## 3. ABA Signaling under Stress

### 3.1. Calcium Signaling Integration with ABA Signaling Pathway and Stomatal Regulation

In plants, abiotic stress positively triggers the levels of ABA and reactive oxygen species (ROS) [75] such as H_2_O_2_ [76,77,78,79]. High H_2_O_2_ levels trigger cytosolic calcium concentration via nitric oxide (NO) [80,81]. Downstream signaling cascades regulate transcriptional responses to abiotic stress tolerance and stomatal regulation (Figure 1B–D) [53]. Many reports point to direct interactions between ABA and calcium signaling systems at different levels (Table 1). For such interactions between ABA and calcium signaling, ABI1 (clade A protein phosphatases 2Cs) appear to function as master regulators [53,58,82]. In normal growth conditions (basal ABA level), Ca^2+^ and SnRK2/3/6/7/8/CDPK activity are inhibited by ABI1/PP2C. This inhibition prevents downstream signaling [83,84] (Figure 1A). In the presence of ABA (during stress or developmental stages), ABI1/PP2C activity is inhibited by ABA, which induces RCARs and elevated levels of H_2_O_2_, and in turn the conversion of ABA signals into appropriate cellular responses where SnRKs (2/3/6/7/8)/CDPK phosphorylate the downstream targets [53]. This is the classical ABA signaling pathway; however, recent findings have suggested that it is not that simple. It is potentially integrated with multiple signaling pathways, such as the calcium pathway. Ca^2+^, along with ABA, represents a most versatile secondary messenger in eukaryotes and is involved in crucial aspects of signaling [85,86,87,88]. Stress signals that trigger cellular ABA levels can also invoke prominent cellular Ca^2+^ signals in plants, which are perceived downstream by calcineurin B-like proteins (CBLs)/CBL-interacting protein kinases (CIPKs) [89,90] (Figure 1B). The calcium sensor CBL-CIPK regulates a variety of downstream targets, such as regulation of stomata and ion channels [91,92,93]. Ca^2+^- dependent protein kinases (CDPKs) have functions similar to those of CBL/CIPKs in ABA signaling [84,94,95,96,97]. OPEN STOMATA 1 (OST1), an SnRK2 protein, has been reported as functioning as a critical regulator in the ABA signaling module [98]. The *ost1* mutant displays a stomatal closure defect under drought stress. Positional cloning of OST1 revealed its similarity to SnRK2.6 [99]. The *ost1*/*snrk2.6* double mutant affects stomatal closure under both stress-driven ABA signaling and normal growth conditions. The ABA signaling pathway is regulated by the direct interaction of SnRK2.6/OST1 and PP2CA/ABI1 (Figure 1C) [100]. 

The target proteins of Ca^2+^-dependent and -independent ABA signaling systems are also the target of other signaling systems. Reactive burst oxidases (RBOHs) are phosphorylated by SnRK2.6/OST1, CPK5, and CBL1-CIPK26 [96,115,116]. At normal ABA levels, SnRK2.6 is inactive, and PP2CA (ABI1) inhibits the S-type anion channel (SLAC1) and the activity of its homologs (SLAH3) [120]. In addition, SnRK2.6 cannot inhibit K^+^ channel (KAT1) activity, which results in increased turgor pressure and stomatal opening [121]. To cope with stress, plants tend to close stomata to prevent water loss. ABA signaling would lead to the closure of stomata. Elevated ABA levels under stress conditions inhibit PP2CA activity and the phosphorylation of SnRKs (Ca^2+^-dependent manner), CBL, CIPK, and CDPK (Ca^2+^-independent manner) occurs leading to the phosphorylation of SLAC1/SLAH3 by CBL1/9-CIPK23, CPK3/6/21/23, and SnRK2.6/OST1 [84,95,118,122,123,124,125]. SnRK2.6/OST1 also inhibits K^+^ channel (KAT1) activity [126] and mediates the efflux of anions and influx of K^+^ and decrease in turgor pressure that results in stomatal closure (Figure 1C).

### 3.2. Abiotic Stress Signaling Integration with the ABA Signaling Pathway

In plants, ABA signaling is an important tool for robust stress responses to environmental stimuli and developmental processes. Plants encounter numerous abiotic stress factors, such as water scarcity (drought or dehydration), low temperature (cold stress), and salinity (salt stress) [59,127]. The plant utilizes ABA to assess the stress impact and may continuously alter ABA signaling stages based on environmental and physiological conditions to delay processes, such as germination, development, and lateral root formation, as appropriate [128]. Under stress conditions, numerous genes are upregulated in plants via the ABA pathway. Promoter analysis of the ABA-inducible genes has indicated that they must have multiple *cis*-elements, such as ABREs (PyACGTGG/TC) [129,130]. Plant gene expression analyses have revealed conserved ABREs cis-acting elements in dehydration-inducible promoters [131]. Sequences of ABREs are also present in the genes that are expressed in the seeds (Figure 1D) [132].

The bZIP subfamily members (AREB1/ABF2, AREB2/ABF4, and ABF3) are induced by ABA, dehydration, and high salinity [133], and the overexpression of the above factors in transgenic plants has led to drought tolerance [133,134,135]. To establish the role of such AREB/ABF TFs in stress-responses in vegetative tissues, Yoshida et al. [136] generated an *areb1*/*areb2*/*abf3* triple mutant. Microarray analysis revealed impaired stress-responsive gene expression. It also revealed many stress-responsive genes, such as LEA proteins, group A PP2Cs, and various types of TFs that lie downstream of AREB/ABF TFs. Most of such gene promoters contain ABRE sequences. The *areb1*/*areb2*/*abf3* triple mutant was sensitive to drought-stress and was more resistant to ABA (primary root growth) when compared with other single and double mutants, suggesting that ABF3, AREB1, and AREB2 are the master TFs that regulate the ABRE-dependent gene expression under stress conditions in ABA signaling. HD-ZIP transcription factor (TF), HAT1, a critical regulator in brassinosteroid (BR) signaling, interacts with SnRK2s [137,138]. HAT1 suppresses ABA signaling and is involved in ABA regulation of drought response [138], which also suggests the integration of BR signaling with ABA signaling to regulate the downstream targets of abiotic stress tolerance.

Mitogen-activated protein kinases (MAPKs) are also involved in ABA signaling in response to abiotic stress [57,139]. Studies on MAPK inhibitors highlighted the link between ABA and MAPK signaling. For example, in barley, phenyl arsine oxide inhibited ABA-induced MAPK activation [140]. Apart from MPK3, MPK4, and MPK6, the only other MAPKs activated in response to ABA are MPK12 [141,142,143] and the C-clade MAPKs MPK1/2/7/14 [144,145,146]. In *Arabidopsis*, MAP3K17 and MAP3K18 function upstream of the MAP3Ks to activate MKK3 and MAP2K, and, therefore, the C-clade MAPKs (MPK1, MPK2, MPK7, and MPK14) in response to ABA signaling (Figure 1D) [82,146,147]. BiFC and yeast 2-hybrid techniques have been used to demonstrate the interactions between kinases in *Nicotiana benthamiana* [146,147]. In the *mkk3* and *map3k17*/*18* backgrounds, ABA-driven activation of MPK7 was significantly reduced [146]. Genetic analysis revealed that in ABA signaling, PYR/PYL/RCAR-SnRK2-PP2C (an ABA core signaling module) activates the MAP3K17/18-MKK3-MPK1/2/7/14 cascade through the transcriptional regulation of MAP3K17/18 followed by MAP3K activation [146,148]. MAP3K18 is also regulated directly by the PYR/PYL/RCAR-SnRK2-PP2C module, suggesting that PP2C phosphatase ABI1 interacts directly with MAP3K18 [82] (Figure 1D). MAP3K18 also controls *RD29B* and *RAB18* expression, two known ABA and abiotic stress-responsive genes, indicating the role of ABRE genes downstream of the MAPK cascade for the ABA signaling-driven abiotic stress response in plants.

### 3.3. Biotic Stress Signaling Integration with the ABA Signaling Pathway

Plants respond to biotic and abiotic stress via crosstalk signals such as ABA, salicylic acid (SA)/jasmonic acid (JA)/ethylene (ET)-mediated defense signaling [149]. The role of ABA in the crosstalk between biotic and abiotic stress is very broad and is discussed in detail by recently published reviews [150,151]. A restraint function of ABA on the systemic acquired resistance pathway of SA induction has also been reported in tobacco [152]. Elicitors/effectors secreted by *Pseudomonas syringae* pv. tomato activate ABA biosynthesis along with ABA signaling, which leads to the inhibition of biotic defense responses [23]. However, several reports have shown the positive effect of ABA signaling on biotic and abiotic stress. For example, treatment with ABA and SA resulted in a short-term increase in H_2_O_2_ production, which induced tolerance to salinity, heat, and oxidative stress [153]. During infection in plants, stomata can act as passive passage for bacteria. *P. syringae* pv. tomato pathogen-associated molecular patterns (PAMPs) induce stomatal closure via ABA signaling, NO production, and flagellin receptor (FLS2), indicating the integration of biotic and abiotic signaling with ABA signaling in the regulation of stomata [154]. β-aminobutyric acid (BABA), a non-protein amino acid, has been reported as a link between heat tolerance, biotic stress, and ABA signaling. Plants treated with BABA become resistant to abiotic as well as biotic stress [155,156,157,158]. The *ibs3*, a BABA-induced sterility mutant, exhibits defected regulation of *ABA1*, salt resistance, and BABA-induced pathogen [159]. A recent study described BABA as a natural molecule synthesized by plants under stress [160]. Therefore, it may be a new entry into the list of plant hormones. Isolation of an activation-tagged mutant of activated disease resistance 1 (*adr1*) further consolidates the link between ABA-mediated biotic and abiotic signaling. The *adr1* mutant displayed drought tolerance as well as disease resistance. Surprisingly, *adr1* plants display sensitive phenotype toward salt and heat stress, suggesting antagonism between biotic stress and abiotic stress [161]. Recently, a study reported that PUB10 acts as a negative regulator of ABA signaling, which could also be intermediatory in JA signaling (Figure 2) [66]. MAPKs are also reportedly involved in plant defense response by regulating the JA- and SA signaling as well as downstreaming transcription factors. This is discussed in detail by a recently published review [162].

In *Arabidopsis*, the biotic stress-inducible AP2/ERF TF family proteins are associated with different abiotic stresses, such as cold, drought, salinity, heat, and light stress [70,163,164,165]. Many ROS-inducible genes are also induced by *AtERF6* for protection against both biotic and abiotic stress [166]. Most of the ethylene response factors (ERFs) that display abiotic stress tolerance are induced not only by ethylene but also by other biotic stress associated phytohormones, such as JA and SA. Therefore, there is potential crosstalk between abiotic and biotic stress and responses via the ABA signaling pathway [167,168,169,170] (Figure 2).

## 4. ABA Signaling in Plant Development

### 4.1. Role of ABA Signaling in Seed Germination and Lateral Root Formation

ABA accumulates during seed development and seed germination. In mature seeds, ABA promotes the synthesis of LEA (Late embryogenesis abundant) proteins for desiccation tolerance. ABA also inhibits germination and stimulates dormancy in mature seeds [55]. *ABI3* and *ABI4* control seed sensitivity and embryonic gene expression in plants [171]. *abi3* mutant seeds display reduced dormancy and vivipary, caused by the strongest alleles. To control seed maturation, ABI3/VP1 binds directly to the promoters of Sph/RY. *FUSCA3* (*FUS3*) and *LEAFY COTYLEDON 2* (*LEC2*) genes encode TFs that are structurally related to VP1/ABI3 [172,173], and the genes interact with ABI5 [174], although VP1/ABI3 is involved directly in ABA signaling. A bZIP protein, ABA-INSENSITIVE5 (AB15), was identified via ABA insensitive germination screening [171]. In addition to ABI5, three AREB/ABF-type bZIP proteins, namely EEL, AREB3, and AtbZIP67/AtDPBF2, are expressed in the nuclei of developing seeds and play vital roles in seed germination [175,176]. During early germination and seed maturation under stress conditions, ABI5 regulates the direct expression of *AtEm1* and *AtEm6* (LEA class genes) [130,175,177]. A seed expressed gene, *DELAY OF GERMINATION 1* (*DOG1*), is critical for dormancy induction. During *Arabidopsis* seed development, *DOG1* interacts with *ABI3* and influences *ABI5* expression [178] (Figure 1E). *PGIP1* and *PGIP2* are associated with the process of seed germination, and they are direct targets of ABI5 [179,180]. Overall, all the above studies highlight the key role of ABI5 as a master regulator of seed development through the ABA signaling pathway. A negative regulator of lateral root formation, MYB96, activates the expression of *ABI5* and is involved in plant responses to salt and drought stress [181]. MYB7 also negatively regulates *ABI5* expression in seeds [182] (Figure 1E). The above studies support the functional role of ABI5 in the ABA signaling pathway-dependent inhibition of lateral root growth under stress conditions [183].

### 4.2. ABA and Light Signaling Convergence

ABA and light are the endogenous hormonal and the external environmental cues that play vital roles in the regulation of seed germination and seed development. The ability of plants to integrate external signals with internal regulatory pathways is crucial for their survival [184,185]. However, the crosstalk between ABA signaling and light signaling and its underlying molecular mechanisms remain largely unclear. The involvement of TF HY5 in promoting seedling photomorphogenesis, root development, and early seedling growth has been studied extensively. It mediates ABA signaling responses in seed germination by binding directly to the *ABI5* promoter and regulating its expression [186]. Two major TFs in the phytochrome A pathway, FAR-RED IMPAIRED RESPONSE1 (FAR1) and FAR-RED ELONGATED HYPOCOTYL3 (FHY3), positively regulate ABA signaling by inducing *ABI5* expression directly [187]. PIL5 (also known as PIF1), a phytochrome-interacting bHLH TF, also targets *ABI5* [188]. Conversely, BBX21, a transcriptional regulator that is involved in the regulation of seedling photomorphogenesis, negatively regulates *ABI5* expression by intervening in the binding of HY5 to the *ABI5* promoter [189]. In addition, ABI5 can regulate its own expression while BBX21 inhibits ABI5 activation (Figure 1E). BBX21 represses ABI5 activity by regulating the binding activities of both ABI5 and HY5 to the *ABI5* promoter [189]. The findings suggest that, in the light signaling pathway, multiple TFs regulate *ABI5* expression in the ABA signaling responses.

### 4.3. ABA Signaling and Control of Flowering Time

A variety of ABA signaling activities are involved in controlling meristem function or flowering time [171,190]. In addition, the ABA inhibitory effect in floral transition was described very well in a study on an ABA-deficient mutant [191]. Such an inhibitory effect could be due to the modulation of DELLA protein activity [184]. Therefore, ABA is also considered a floral repressor. FLOWERING LOCUS C (FLC) is a key repressor integrator that tightly controls flowering signals [192]. FLC also mediates seed germination via genes, such as *SOC1*, *APETALA1*, and *FT*, making FLC an effective regulator in temperature-dependent seed germination [193]. ABFs are the bZIP TFs that are involved in ABA signaling during seed germination in plants [194,195]. Another bZIP protein, FD, mediates signals from FT at the shoot apex [196]. Overexpression of another bZIP TF, *ABI5*, upregulates FLC expression and delays flowering initiation. Phosphorylation of ABI5/SnRK2 during ABA signaling directly affects floral transition, and the inhibitory effect of ABI5 on floral transition disappears without phosphorylation. Transactivation of FLC expression could occur by direct binding of ABI5 to *FLC* promoter regions [197]. AtU2AF65b, a putative U2AF65 spliceosome, participates in ABA-mediated flowering via the regulation of the pre-mRNA splicing of *ABI5* and *FLC* [198], which indicates the positive regulation of FLC activity by ABI5 during ABA signaling. Furthermore, AtU2AF65b-mediated mRNA splicing is critical for ABA-regulated flowering transition for the control of floral transition in plants (Figure 1E).

## 5. Other Aspects of ABA Signaling

ABA transporters are also a significant part of ABA signaling, as it is important to transport ABA from its sites of synthesis to its multiple sites of action within plants. In *Arabidopsis*, four ABA transporters have been identified (AtABCG25, AtABCG30, AtABCG31, and AtABCG40) all of which are ATP-binding cassette transporter G subfamily members [199,200,201,202]. AtABCG25 is involved in exporting ABA from the vasculature [201], while AtABCG40 is a plasma-membrane ABA-uptake transporter in guard cells, and is necessary for timely closure of stomata in response to drought stress and seed germination [199,200]. AtABCG30 mediates ABA uptake into the embryo, while AtABCG31 brings about ABA secretion from the endosperm [200]. A recent study reported *ABA transporter-like 1* (*AhATL1*) gene from peanut (*Arachis hypogaea* L.) whose cognate protein, AhATL1, is a member of the ATP-binding cassette transporter G subfamily and localizes to the plasma membrane [203]. The expression of both the AhATL1 transcript and the corresponding protein was upregulated by water stress and treatment with exogenous ABA. Another report suggested that in *Medicago truncatula*, MtABCG20 acts as an ABA exporter that influences root morphology and seed germination [204]. These data indicate that the ABA transport system plays a significant role in water deficit tolerance and growth regulation [203].

ABA signaling crosstalk occurs with other hormones that are involved in plant growth and stress response. These hormones include strigolactone, cytokinin, and karrikin. Strigolactone (SL) is a recently discovered class of phytohormone that inhibits shoot branching [205]. ABA signaling may regulate SL biosynthesis [206]. The antagonistic action of ABA and cytokinin signaling mediates drought stress response in *Arabidopsis* [207]. Karrikin signaling pathway seems to be upstream of ABA signaling pathway and karrikin mediates changes in ABA-related gene expression [208]. DELLA protein is important for seed germination [209]. ABA also interacts with DELLA protein when DELLA/ABI3/ABI5 complex is involved in seed germination [210].

## 6. Conclusions

**Figure 3:**
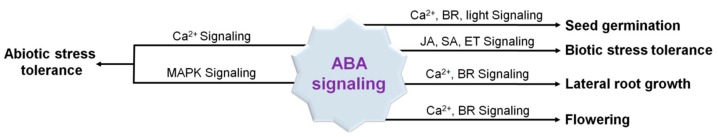
Integration of various signaling pathways with ABA signaling. ABA signaling plays a central role in regulating different developmental processes, including stress responses, as is evident from its interactions with calcium (Ca^2+^), jasmonic acid (JA), salicylic acid (SA), brassinosteroid (BR), ethylene (ET), and MAP kinase (MAPK) signaling pathway members.

It is evident that ABA is an important signaling compound. In stress and developmental responses in plants, ABA signaling largely depends on the SnRK family of protein kinases. ABA signaling integrates other signaling components, such as Ca^2+^, light, MAP kinase, SA, JA, and ET signaling, in response to environmental cues, developmental activities, and biotic stress (Figure 3). Such integration is vital for response stress and plant development; however, there are still gaps regarding to what extent and how often such integrations occur. In addition, it is important to reveal the complex ABA signaling network by adopting more integrated and more detailed genome-wide studies to identify the critical components of stress responses and developmental processes and to develop scientific tools for the genetic engineering of stress-tolerant and robust plants. Furthermore, it is critical to determine the role of all ABA signaling-related genes to fill any knowledge gaps about ABA signaling. In the future, studying the function of ABA signaling-related genes under different combined stress conditions and the regulation of developmental processes would offer detailed insights into the underlying mechanism of ABA signaling.

## Figures and Tables

**Table 1:** List of the target genes that are regulated by ABA as well as Ca^2+^ signaling.

Gene Name	Accession Number	Main Function	Regulated by Ca^2+^-Dependent ABA Signaling	Regulated by Ca^2+^-Independent ABA Signaling	Reference
*ABI5*	AT2G36270	bZIP TF	CIPK11/26, activates by phosphorylation	SnRK2s’ phosphorylation activation; PP2Cs’ dephosphorylated inactivation	[67,101,102]
*ABF1/4*	AT1G49720/ AT3G19290	bZIP TF	CPK4/11, activates by phosphorylation	SnRK2s’ phosphorylation activation; PP2Cs’ dephosphorylated inactivation	[101,103]
*AKT1*	AT2G26650	Potassium ion channel	CBL1/9/CIPK23, activates by phosphorylation	HAI2 and PP2CA, regulate AKT1	[104,105,106]
*AKT2*	AT4G22200	Potassium ion channel	CBL4/CIPK6, localized in the plasma membrane	PP2CA, regulates AKT2	[107,108]
*KAT1*	AT5G46240	Potassium channel	Inhibited by the SnRK2s and involved in the stomatal closure	Inhibition by SnRK2s is inhibited by ABI1, involved in the stomatal opening	[109,110]
*NPF6.3*	AT1G12110	Nitrate transporter	CBL1/9CIPK23, deactivates under high nitrate conditions and increases the nitrate sensitivity	ABI2 involved in the dephosphorylation or deactivation of CBL1/CIPK23	[111,112]
*SLAC1*	AT1G12480	Plasma membrane anion channel	Induced by the SnRK2s and involved in stomatal closure	Induction by SnRK2s is inhibited byABI1, involved in the stomatal opening	[113,114]
*RBOHF*	AT1G64060	Plasma membrane superoxide generation	CBL1/9/IPK26, activates by phosphorylation	OST1 involved in phosphorylation	[115,116]
*RBOHD*	AT5G47910	Plasma membrane superoxide generation	CPK5, activates by phosphorylation	-	[96]
*SnRK2.6/OST1*	AT4G33950	Calcium-independent ABA-activated protein kinase	CBL/CIPL/CDPK, activates by phosphorylation	SnRK2.6 involved in phosphorylation	[112,117]
*SLAH3*	AT5G24030	Anion channel	CBL1/9/CIPK23	ABI1 involved in deactivation	[95,118,119]
			CPK21 involved in phosphorylated activation; CPK21 also recruits SLAH3 onto the membrane		
